# Houston District Medical Society

**Published:** 1895-03

**Authors:** 


### Houston District Medical Society.

Regular quarterly meeting held in Houston, December 31, 1894. Vice-President R. W. Knox, M. D., presiding; Dr. S. C. Red, acting Secretary. The President, Dr. E. T. Cook, and the Secretary, Dr. J. B. Massie, absent on account of sickness.

After transaction of routine business, Dr. G. W. Christian read a paper on "Chronic Invalidism." [Published herewith, the TEXAS MEDICAL JOURNAL being the official organ of the Society.—ED.]

Discussion was opened by Dr. J. W. Scott, who approved of Dr. Christian's plan of treatment. He thought also that the rest-cure and massage offered resources that should not be ignored.

Mr. Morris (Robt. T.) gave it as his opinion that the ætiology of chronic invalidism had been too much neglected. He could not agree with Dr. Christian that there is no lesion. He took as a basis of his remarks that all disease has a "lesion," whether it can be demonstrated or not.

Dr. Bacon thought the term "chronic invalidism" embraces cases with and without pathological lesion. He cited instances from his practice and experience which demonstrated that the complaint in many instances is purely mental.

Dr. F. M. Davis endorsed the paper and recalled some of his experiences which bore out the position taken.

Dr. S. C. Red stated that it had been his practice to manage chronic invalids just the same as children are managed, whose mental faculties have not been fully developed, or invalid adults whose mental faculties are in abeyance or abnormally functionating.

Dr. L. Knox expressed the opinion that the confidence of the patient was not so difficult to acquire as to retain. He looked upon quacks and charlatans as therapeutic agents, especially adapted to this class of cases.

Dr. Renfro spoke from the text "mind rules matter," and related some "hoodoo" experiences.

The talk then drifted into reminiscences, and a chatty and pleasant interchange of experiences and opinions was indulged in for an hour, when, on motion, the Association adjourned to the Burton building, where an excellent banquet was spread. Toasts were offered to the "medical profession," to certain "medical men," to the "ladies," to the "press." The festivities were prolonged till after 12 o'clook. They saw the old year off and the new year in, and vowed and resoluted, then and there, to repeat the performance—as many as are spared—each recurrence of the day, December 31st.

